# Extending conventional surface roughness ISO parameters using topological data analysis for shot peened surfaces

**DOI:** 10.1038/s41598-022-09551-9

**Published:** 2022-04-01

**Authors:** Jan F. Senge, Asghar Heydari Astaraee, Pawel Dłotko, Sara Bagherifard, Wolfram A. Bosbach

**Affiliations:** 1 Department of Mathematics and Computer Science, University of Bremen, 28334 Bremen, Germany; 2 Department of Mechanical Engineering, Politecnico di Milano, 20156 Milan, Italy; 3 Dioscuri Centre for Topological Data Analysis, IMPAN, 00-656 Warszawa, Poland; 4 Diagnostic and Interventional Radiology, University of Heidelberg, 69120 Heidelberg, Germany

**Keywords:** Mechanical engineering, Biomaterials, Theory and computation

## Abstract

The roughness of material surfaces is of greatest relevance for applications. These include wear, friction, fatigue, cytocompatibility, or corrosion resistance. Today’s descriptors of the International Organization for Standardization show varying performance in discriminating surface roughness patterns. We introduce here a set of surface parameters which are extracted from the appropriate persistence diagram with enhanced discrimination power. Using the finite element method implemented in Abaqus Explicit 2019, we modelled American Rolling Mill Company pure iron specimens (volume 1.5 × 1.5 × 1.0 mm^3^) exposed to a shot peening procedure. Surface roughness evaluation after each shot impact and single indents were controlled numerically. Conventional and persistence-based evaluation is implemented in Python code and available as open access supplement. Topological techniques prove helpful in the comparison of different shot peened surface samples. Conventional surface area roughness parameters might struggle in distinguishing different shot peening surface topographies, in particular for coverage values > 69%. Above that range, the calculation of conventional parameters leads to overlapping descriptor values. In contrast, lifetime entropy of persistence diagrams and Betti curves provide novel, discriminative one-dimensional descriptors at all coverage ranges. We compare how conventional parameters and persistence parameters describe surface roughness. Conventional parameters are outperformed. These results highlight how topological techniques might be a promising extension of surface roughness methods.

## Introduction

The quantification of surface roughness by appropriate mathematical description methods is of mandatory importance for a wide range of technical and medical applications. This is true for any surface modification procedure aimed at surface functionalization. For example, in the medical field, bacterial adhesion and the interaction of human cells with surfaces can be modulated by altering surface topography^1,2^ Surface roughness has an important role in defining the mechanical performance of material under cyclic loading. Wear, scratch and corrosion resistance are also substantially affected by surface roughness parameters^3,4^. It is worth mentioning that these properties are not isolated from each other and interdependencies between them exist^5^.

Standardized surface roughness parameters exist and describe a surface by its roughness and wider topography as indicated in standards like 25178^6^ and 4287^7^ by the International Organization for Standardization (ISO), Japanese Industrial Standards (JIS) B 0601^8^ or the German Institute for Standardization (DIN) 4762^9^. Those standards provide a plethora of different surface texture parameters defined on profile-and area-based surfaces^10^. Among these, *conventional surface roughness parameters* assessing the properties of the whole measured surface are the most commonly used, in particular in the field of impact-based surface treatments. They focus on summaries such as the arithmetical mean deviation (Ra, Sa) as well as the root mean square deviation of the surfaces (Rq, Sq). While these two parameters are undoubtedly useful, they are shown to be inappropriate to highlight dissimilarities in topographies obtained by e.g. impact based surface treatments such as shot peening^2,11^; in addition they neglect spatial information about peaks and valleys. While the standards define other parameters exploiting the spatial information, these rely either on the choice of the evaluation length or area, or -in the case of feature-based characterisation- on appropriate use of segmentation of the surface^12^. Area-based topographical characterization has gathered attention in recent years. Either summary statistics are calculated for a surface area or the spatial information is exploited to improve surface characterisation^13^. There exist techniques to extend profile-based parameters to area-based surfaces: Averaging parameter values of all profiles in horizontal and vertical direction, calculating average values over a growing number of directions^14^ or creating surface roughness signatures through hierarchically organized regions^15^. In the analysis, we will focus on the conventional area-based parameters.

*Shot peening* is an established surface modification procedure during which shots are accelerated by a compressed air flow. It is commonly used for enhancing fatigue properties of metallic materials. The shots create multiple impacts on the target surface causing inhomogeneous plastic deformation and thus generating compressive residual stresses that delay crack propagation^16^. Numerical analysis by finite element (FE) method is able to complement experimental research in optimizing the process parameters^17^. In general, peening involves a competition between the beneficial effects on the component’s performance in terms of subsurface compressive residual stresses, surface work-hardening, and surface nanocrytallization versus the side effect of surface roughening (that is not always desired)^18^.

*Topological data analysis* (TDA) is a field in mathematics interested in quantifying and finding out more about the shape of structures^19^. It provides tools for multi-scale analysis of the geometric and topological properties of an object and has proved helpful in a plethora of different applications^20^. Persistent homology^21^ is one of the most prominent techniques of TDA. It is based on the idea that for a suitable real-valued function $f:X\to {\mathbb{R}}$ describing the height of the surface $X$ we can track the evolution of connected components and holes of $f$. This evolution is given by the homology groups of dimension 0 and 1 of the sublevel sets ${f}^{-1} ((-\infty , a])=\{x\in X|f(x)\le a\}$. In other words, the sublevel sets of $f$ give a nested sequence of subspaces, $\varnothing ={X}_{0}\subseteq {X}_{1}\subseteq ...\subseteq {X}_{n}=X$, called filtration. Keeping track of homology of these subspaces gives the persistence diagram of the surface $X$ with the height function $f$. Suitable vectorizations^22–24^ of the persistence diagram provide machine learning algorithms with an additional information about the surface $X$.

In *this present study* we use TDA that is complementary in nature to existing characterizations of surface topography. In particular, based on recent work^25^, we introduce the TDA pipeline for characterizing surface roughness and a novel collection of roughness descriptors. More precisely, we define several *persistence-based surface roughness parameters* and compare their performance to the conventional parameters. The study inputs are modelled surfaces of American Rolling Mill Company (Armco) pure iron (99.89%) samples. The input surfaces are obtained through a set of FE simulations^26^. Impacts by the peening material lead to dents (or dimples) on the material surface. Peening coverage or coverage percentage is defined as the percentage of a given surface area impacted by the peening media^27^. Coverage percentage can be extended to values beyond 100% (full coverage), but it is not part of the present study. The numerical FE simulation provides samples of surfaces of increasing shot peening coverage.

## Materials and methods

Shot peened surfaces are generated by FE^28^ and postprocessed for obtaining the roughness surface, defined by ISO 25178. We calculate surface roughness parameters from the roughness surface. For performance comparison, we add classification and clustering evaluations.

### FE generated dataset and postprocessing

**Figure 1:**
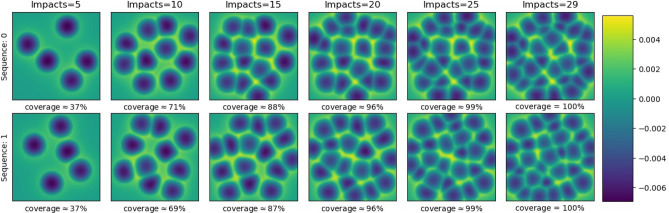
Numerical surface samples, colouring by height of the associated surface, for two sequences with number of impacts and estimated coverage percentages per sample.

The dataset used for this study consists of a total of 92 samples, Table 1. Each sample is a surface representation obtained under the shot peening treatment; Armco pure iron, sample volume 1.5 × 1.5 × 1.0 mm^3^. The FE model has been published and experimentally validated in^28^ for carburizing steel. It has been applied in^25^ for Armco pure iron. Each run of the FE model produces one sequence of sample surfaces, Fig. 1. A sequence starts with an untreated plane surface and adds shot peening impacts. Each sequence yields between 6 and 7 surface samples of increasing shot peening coverage. Each surface sample is a surface snapshot during the FE run labelled by one of 6 stages.

**Table 1:** Dataset obtained from the FE code^28^, class labels assigned by coverage percentage.

Stage	1	2	3	4	5	6	7	Total
Range for the number of impacts	4–5	9–10	14–15	19–20	24–25	27–30	32–33	–
Number of samples	15	15	15	15	15	15	2	92
Minimum coverage	30.49%	63.33%	84.16%	94.02%	99.02%	99.87%		–
Mean coverage	36.75%	68.95%	88,27%	96.48%	99.43%	99.99%		–
Maximum coverage	37.97%	71.63%	90.70%	97.82%	99.84%	100%		–
Bin	0	1	2	3	4	5		–
Class labels	37%	69%	88%	96%	99%	100%		–

The stages at which surface snapshots are taken by the FE code correspond to ranges of impact numbers, Table 1. Based on the impacts, we calculate the shot peening coverage of the surface. In this calculation, we locate the local surface minima on the height displacement map which represent an estimation of shot impact centres. Each shot is estimated to shot peen a surface circle of diameter 0.055 mm. To estimate the coverage percentage, the macro circle of diameter 0.2 mm is considered. We assign each of the 92 samples, according to estimated sample coverage, to one of 6 bins. Bins 0 to 4 contain 15 samples each. The 5th bin contains 17 samples. The bins are labelled using the mean coverage percentages of each bin, this will be the class label for the analysis.

Each individual FE surface sample is given on a regular, unevenly spaced 74 × 74 surface grid, but different grids may be slightly shifted with respect to each other. Hence, for comparison purposes, all the values are interpolated on a common 74 × 74 grid being the input for further analysis. For calculating the ISO 25178 parameters, the interpolated FE surfaces are postprocessed to give a roughness surface by applying a low-pass Gaussian filter as L-filter (utilizing Fast Fourier transformation) for the cut-off wavelength of 0.8 mm, see ISO 16610:61^29^. Due to the construction of the numerical model used, applying an S-filter is not required.

### Persistent homology

**Figure 2:**
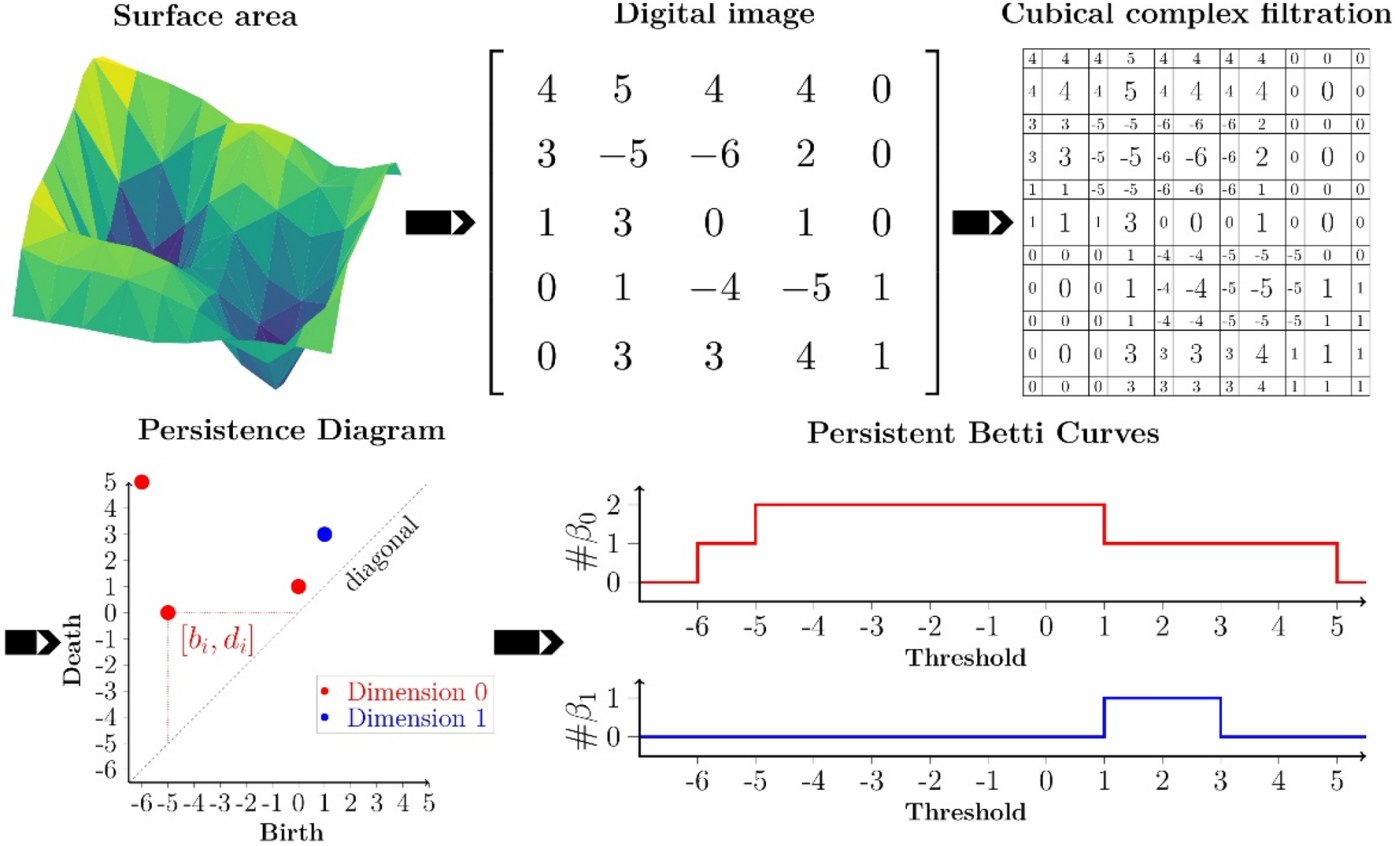
Pipeline for the calculation of persistent diagrams and Betti curves (BC) in dimension 0 and 1: the result of the FE simulation is interpolated on a regular grid to obtain a digital image; the pixels of which are converted to top-dimensional cells of a two-dimensional cubical complex. We do a sweep of the sublevel sets of this complex keeping track at which height values the connected components and holes change. As a result, we obtain the birth and death values of points in the persistent diagram in dimension 0 and 1. Counting the number of connected components and holes for different thresholds in the filtration gives us the BC.

As mentioned above, persistent homology is one of the most prominent techniques used in TDA^19,21^. It captures the changes in homology, that is how connected components and holes of a filtration evolves. In the following we explain the pipeline shown in Fig. 2 for computing persistence diagrams in the case of the surfaces we encounter in this study.

Given data points as height values of a surface, we transform it to a digital grayscale image by interpolation to an evenly spaced grid.

A suitable underlying combinatorial structure of the image allowing for efficient representation and computation of homology are cubical complexes^30^ described below.

A two-dimensional cubical complex consists of elementary $d$-cubes $\sigma \subset {\mathbb{R}}^{2}$ for $d\in \{\mathrm{0,1},2\}$ which are products of two elementary intervals $\sigma ={e}_{1}\times {e}_{2}$ of the form $[k,l]$ for $l\in \{k,k+1\}$ and integers $k$. Hence, a two-dimensional cubical complex consists of vertices represented as $[k,k]\times [l,l]$, edges being either of the form [$k,k+1]\times [l,l]$ or $[k,k]\times [l,l+1]$ and squares $[k,k+1]\times [l,l+1]$ for integers $k$, $l$. Having constructed a cubical complex for the image, we now need to assign the values of the pixels to the elementary cubes of the cubical complex. There are two main constructions which differ in their assignment of the pixels. The pixel value of an image is assigned to either the vertices or the top-dimensional cubes, which in the case of a two-dimensional cubical complex are squares^31^. In this present study, we follow the second approach, and use the library of the Geometry Understanding in Higher Dimensions (GUDHI) project^32^ for the efficient computation of cubical complexes and their persistent homology.

Having constructed the associated cubical complex for the image, we now consider its sublevel set filtration obtained from values of the top-dimensional cells and its corresponding persistence diagrams. A point ${(b}_{\alpha }, {d}_{\alpha })$ of a diagram in dimension k is characterized by the birth time ${b}_{\alpha }$ and death time ${d}_{\alpha }$ for a k-dimensional homology class $\alpha$. A persistence diagram consists of a multiset of points in the extended plane^19^ i.e. a set of tuples in ${\mathbb{R}}\cup \{\mathbb{\infty }\}$ where the same tuple can occur multiple times.

The value ${d}_{\alpha }- {b}_{\alpha }$ is called persistence of the homology class $\alpha$. Due to the process of computing the persistence diagrams from digital images, there will only be a finite number of points in the output diagram. In the following analysis we will only consider persistence diagrams in dimension zero (0D) and one (1D).

We can define several different distances between persistence diagrams. Most common are the bottleneck and Wasserstein distance^33^. Diagrams, with those distances, are stable with respect to bounded perturbation of the input surface. Hence, the space of diagrams is a metric space. On the other hand, using statistical analysis on persistence diagrams is challenging as, for example, they do not have a unique mean^34^. Certain vectorizations and summaries of persistence diagrams have been developed to overcome these shortcomings^22^. These vectorizations are used to construct surface descriptors. We call them *persistence-based roughness parameters* and will specify them in the next section. Further analyses of the persistence diagram are possible^35^.

### Surface roughness parameters

Implemented surface roughness parameters of this present study are shown in Tables 2 and 3. In the analysis of surface roughness, we focus on area-based surface roughness parameters corresponding to the surface texture and topography specification provided in ISO 25178. We select the most prominent parameters for the comparison following the assessment in^25^. The selection includes area height parameters focusing on the vertical distances to a reference plane of the shot peened surfaces as well as hybrid parameters.

**Table 2:** Overview of implemented conventional surface roughness parameters for ordinate values $z(x,y$) within a definition area (A), equations as in ISO 25178.

Name	Symbol	Equation
Arithmetical mean height (µm)	${S}_{a}$	$\frac{1}{A}{\iint }_{A}|z(x,y)| dxdy$
Root mean square height (µm)	${S}_{q}$	$\sqrt{\frac{1}{A}{\iint }_{A}{z}^{2}(x,y) dxdy}$
Skewness	${S}_{sk}$	$\frac{1}{{S}_{q}^{3}}\frac{1}{A}{\iint }_{A}{z}^{3}(x,y) dxdy$
Kurtosis	${S}_{ku}$	$\frac{1}{{S}_{q}^{4}}\frac{1}{A}{\iint }_{A}{z}^{4}(x,y) dxdy$
Maximum peak to valley height (µm)	${S}_{z}$	$\underset{x,y\in A}{\mathrm{max}}z(x,y)-\underset{x,y\in A}{\mathrm{min}}z(x,y)$
Root mean square gradient	${S}_{dq}$	$\sqrt{\frac{1}{A}{\iint }_{A}\left[{\left(\frac{\partial z(x,y)}{\partial x}\right)}^{2}+{\left(\frac{\partial z(x,y)}{\partial y}\right)}^{2}\right] dxdy}$
Developed interfacial area ratio	${S}_{dr}$	$\frac{1}{A}\left[{\iint }_{A}\left(\sqrt{\left[1+{\left(\frac{\partial z(x,y)}{\partial x}\right)}^{2}+{\left(\frac{\partial z(x,y)}{\partial y}\right)}^{2}\right]}-1\right)dxdy\right]$

**Table 3:** Persistent parameters for a k-dimensional diagram ${D}_{k}= \left\{({b}_{i},{d}_{i})\right\}$ for $i=1, ...,m$.

Name	Symbol	Equation
Number of (off-diagonal) points	$n={n}_{{D}_{k}}$	$\mathrm{cardinality}\left\{(b, d)\in {D}_{k}| b<d\right\}$
Persistence of a pair $({b}_{i},{d}_{i})$	${l}_{i}={{l}_{i}}^{{D}_{k}}$	${d}_{i}-{b}_{i}$
Sum of persistence	$L={L}_{{D}_{k}}$	$\sum_{i=1}^{n}({d}_{i}-{b}_{i})$
Average persistence	$\overline{L }={\overline{L} }_{{D}_{k}}$	$\frac{1}{n}\sum_{i=1}^{n}({d}_{i}-{b}_{i})$
Persistence entropy^23^	$E({D}_{k})$	$-\sum_{i=1}^{n}\frac{{l}_{i}}{L}\mathrm{log}\left(\frac{{l}_{i}}{L}\right)$
Persistence Betti curve (BC)^36^	${{\beta }_{k}(s) = \beta }_{{D}_{k}}(s)$	${\beta }_{{D}_{k}}\left(s\right):{\mathbb{R}}\to {\mathbb{N}},s\mapsto \mathrm{cardinality}\left\{(b, d)\in {D}_{k}| b\le s<d\right\}$
Persistence landscape (LS)^22^	$\lambda$	$\lambda :{\mathbb{N}}\times {\mathbb{R}}\to \left[0,\infty \right],$ $\lambda (m,t)=m$-largest value of ${\{{f}_{i}(t)\}}_{i=1}^{n}$ for ${f}_{i}(t) = \underset{}{\mathrm{max}}\left\{\underset{}{\mathrm{min}}\left\{t-{b}_{i}, {d}_{i}-t \right\}, 0\right\}$
Persistence silhouette (Si)^37^	S	$S:{\mathbb{R}}\to {\mathbb{R}}$, $S(t) = \frac{\sum_{i=1}^{n}{w}_{i}{f}_{i}(t)}{\sum_{i=1}^{n}{w}_{i}}$ ${\mathrm{for} w}_{i}= \left|{d}_{i}-{b}_{i}\right|$

Since the persistence diagram is a multiset, the same persistence pair can occur several times in summations and sets. For simplicity sake, we often drop the index $k$. The death time of the infinite persistence pair in dimension 0 is changed to the maximum height value of the roughness surface.

The ordinate value $z(x,y)$ is defined in ISO 25178 as the height of the roughness surface at a specified position $(x,y)$ according to the reference surface. For the calculation of the parameters, the reference surface is chosen to be the mean height plane ie the plane of constant z-value equal to the mean height of the input surface.

Persistent homology offers several enhancements to the above conventional parameters, like stability properties as well as the ability to extract persistence diagrams by using different filtrations. We extract the features from a persistence diagram in dimension k of the form ${\mathrm{D}}_{\mathrm{k}}= \left\{({\mathrm{b}}_{\mathrm{i}},{\mathrm{d}}_{\mathrm{i}})\right\}$ for $\mathrm{j}\in 1, ...,\mathrm{m}$. ${D}_{k}$ is a multiset and can contain the same persistence pair multiple times. As shown in Table 3, persistent entropy^23^ follows the definition of entropy introduced by Shannon^38^. Shannon entropy is defined by $-{\sum }_{i=1}^{n}{p}_{i} \mathrm{log}\left({p}_{i}\right)$ where ${p}_{i}$ are values in the interval $\left[\mathrm{0,1}\right]$, and give the average level of information or surprise of the possible outcomes of a random variable. For persistent entropy, we use the lifetime entropy ${p}_{i}={l}_{i} / L$ and the natural logarithm.

k-dimensional Betti curves (BC)^36^ are 1D functions counting the k-dimensional Betti numbers at different levels of filtration. BC and persistence diagrams are higher dimensional descriptors that can be transformed into 1D descriptors by calculating an appropriate mathematical norm of them. The norm can be understood in this context as the distance of the descriptor of the surface to the descriptor of a roughness surface with constant amplitude of 0.

Persistence landscapes are, like BC, functional summaries of persistence diagrams. A landscape is a sequence of functions ${\lambda }_{m}$ for $m=0, 1, ...$ as defined in^22^. The persistence silhouette^37^ is a smoothed version of the landscape. It transforms a persistence diagram into the vector space of continuous real-valued functions on ${\mathbb{R}}$ by combining all landscapes of different orders into a weighted average function. See Table 3 for the formulas.

Since the surfaces are covered with dimples and the characteristics we want to extract are related to coverage, the 0D persistent diagram encodes most of the relevant information for the current data. Therefore, we limit ourselves to the case of 0D homology in the following analysis.

### Classification and clustering

The two main comparisons for the performance of the conventional and persistence-based parameters are a supervised classification task and an unsupervised clustering task.

For the classification task, the multi-class accuracy under different classifiers is evaluated. Multi-class accuracy^39^ in the context of machine learning is based on the confusion matrix for the classifier and computes the proportion of correctly classified samples to those that are not correctly assigned. In addition to accuracy, the macro-averaged F1 score^40^, which is the mean of precision and recall score for each class, is calculated. We execute a 50/50 split into training set and test set of the 15 shot peening sequences and a tenfold cross-validation.

The clustering relies on a TDA clustering algorithm called ToMATo (Topological Mode Analysis Tool)^41^, implemented in the GUDHI library. This clustering procedure enhances a graph-based hill climbing algorithm with persistence diagrams. It can obtain clustering results without prior knowledge of the number of coverage labels in the data. The clustering is evaluated by a confusion matrix.

Depending on the kind of input, we combine different classifiers with different hyperparameters, see Table 4. We choose the best performing of these hyperparameters for each type of classifier. For the case of scalar features like the conventional roughness parameters and scalar persistence-based parameters, we consider either multiple features or individual parameters as the sole feature. The classifiers used in these cases are Support Vector Machine (SVM) for polynomial, radial basis function and sigmoid kernel, Decision Trees (DT), Random Forests (RF), Multi-layer Perceptron (MLP), as well as a k-nearest neighbours vote (KN) as implemented in^42^.

**Table 4:** Classification pipeline for different inputs.

Input type	Input features	Number features	Scaling	Dimension reduction	Classifiers
Collection of scalar features	All conventional parameters, Table 2	8	Standard-scaling Min–max-scaling to [0,1]	None PCA LDA	Support Vector Machine (SVM) with polynomial, radial basis function, sigmoid kernel Decision tree (DT) Random forest (RF) Multi-layer perceptron (MLP) k-nearest neighbours vote (KN)
	Skewness and developed interfacial area ratio	2			
	0D entropy, maximum 0D persistence, and ${L}^{2}$-norm of 0D BD, LS, Si	5			
Single scalar feature	A conventional or persistence-based parameter	1			
Persistence diagrams	Betti curve (BC)	100	None	None	SVM RF
	Persistence landscapes (LS)	100, 200	None	None	
	Silhouettes (Si)	100, 200	None	None	

To use persistence diagrams as the input for classification, we consider higher order vectorizations of the diagrams, namely BC, persistence landscapes, and silhouettes sampled on 100 or 200 points. The classifiers used in these cases are RF and SVM.

Normalizing or standardizing of parameters ensures compatibility of different features when they are combined. In the case of the multiple scalar features, Principal Component Analysis (PCA) or Linear Discriminant Analysis (LDA) lead to improvements in the analyses^43^.

## Results and discussion

In the following, we compare the conventional roughness parameters and the persistence-based parameters for the dataset of 92 samples of the FE modelling processed and postprocessed as described in the “Method” section.

**Figure 3:**
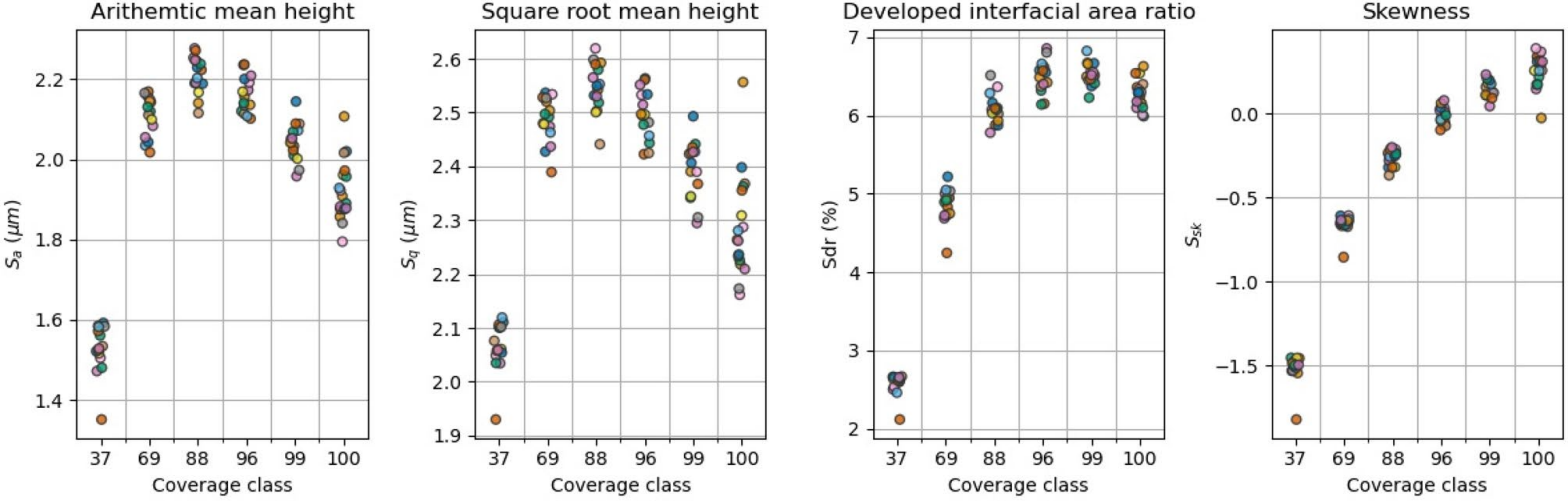
The conventional surface roughness parameters over coverage, colouring by impact sequence. Points marginally shifted in x-direction for better visibility.

Figure 3 shows the conventional surface roughness parameters plotted over the six coverage classes. The arithmetic mean height (Sa) and the square root mean height (Sq) fail to distinguish between samples from different coverage classes due to overlap of y-values for coverage above 69%. Furthermore, there is a considerable y-spread. The developed interfacial area ratio (Sdr) shows a slightly better result in terms of y-spread and y-overlap for coverage up to 88%. However, y-value ranges still overlap for greater coverage classes. Skewness (Ssk) shows the best performance of all conventional parameters which we consider in this present study. Only small overlap of the y-ranges is obtained. For increasing coverage, a clear trend is visible.

**Figure 4:**
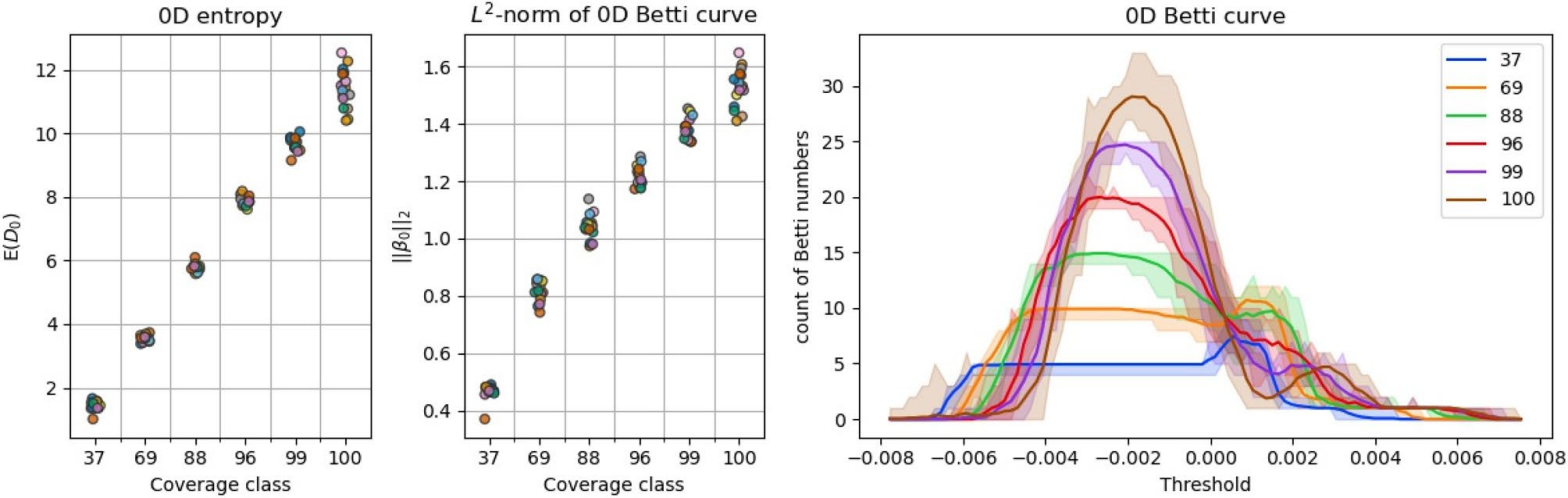
The persistence-based surface roughness parameters over coverage, colouring by impact sequence. Points marginally shifted in x-direction for better visibility. In addition, 0D BC over threshold value (lines = mean, shading = minimal and maximal function values).

Figure 4 shows scatterplots for the 0D persistence entropy and the ${\mathrm{L}}^{2}$-norm of 0D BC, as well as 0D BC. In both scatterplots, we obtained a good approximation of a linear relationship over coverage up to 100% compared to the clearly non-linear trends for the other descriptors. Y-spread is greatest in both cases for 100% coverage. There is a small y-overlap in the results for the ${\mathrm{L}}^{2}$-norm values between coverage classes; in the case of the 0D persistence entropy, there is no y-overlap. The lines plotted in the vectorization of the 0D BC are the mean of each coverage class. In addition, shading is done to visualize the minimal and maximal function values for each threshold considering all BC belonging to the same coverage class. The curves thus symbolize the evolution of the number of connected components during the increase of the height threshold. In the beginning the number of components increases when the threshold reaches the lowest points of each dimple. During this phase may be regarded as a single, separated pit. Exceptions can occur depending on the height of the ridges separating the different pits. The Betti numbers increase until we reach a threshold where connected components are joined together. This starts at roughly −0.002. This value represents in this case the best threshold for separating the classes and could be used for a further analysis. In our case we use the Betti curves whole. From the threshold value 0 onwards the Betti numbers increase a second time. This happens due to the fact, that connected components are created at the borders of the numerical surface samples at that point which are still separated from the large, connected component for the pits in the center by higher ridges on the outside which were not influenced by other shots hitting the vicinity. As mentioned, the value of the 0D BC for x < 0 reaches one plateau or maxima in the case of each coverage class. These plateaus show a clear separation of the different coverage classes.

**Figure 5:**
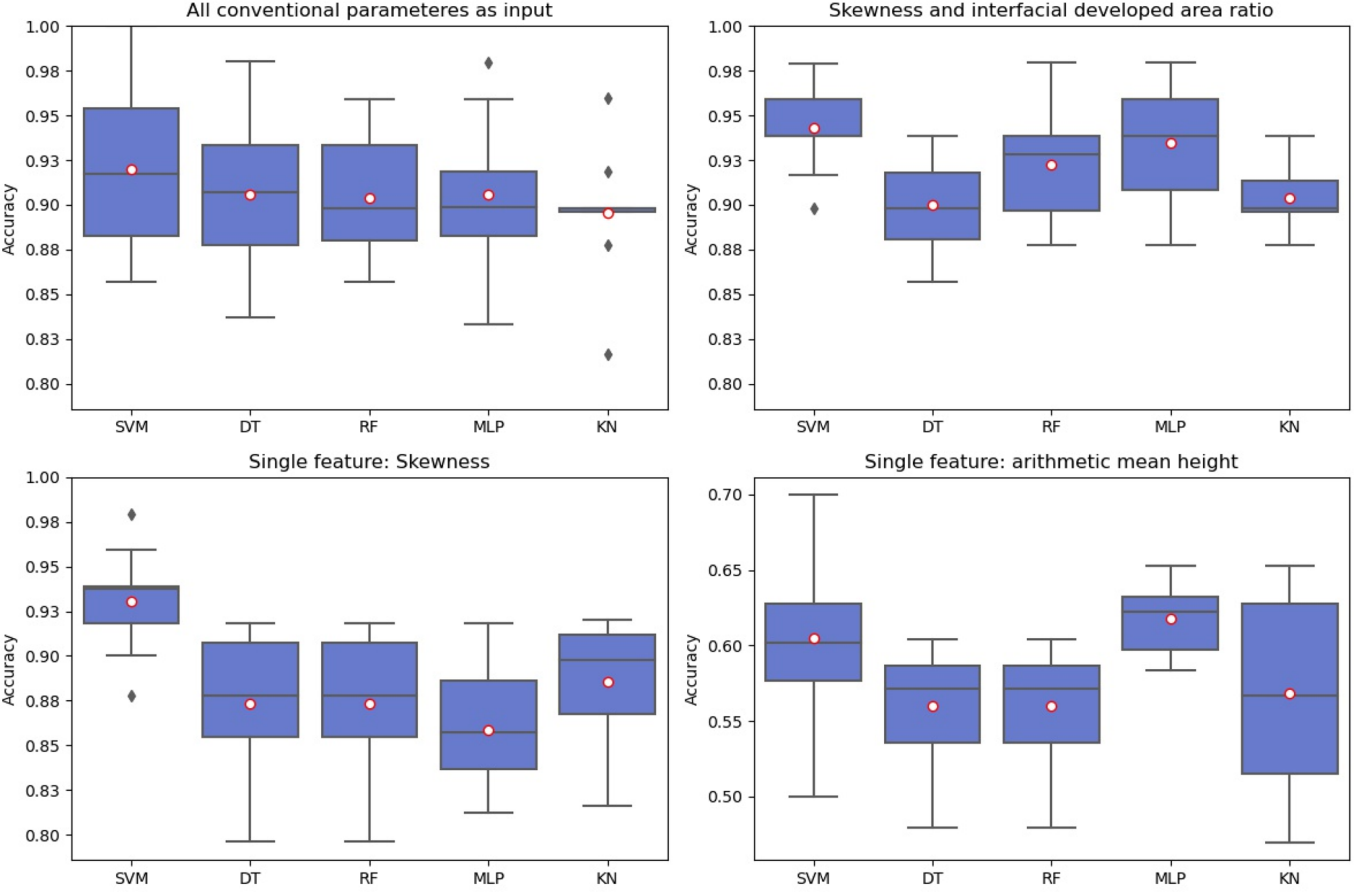
Conventional parameters and classification accuracy obtained for classifiers SVM, DT, RF, MLP, and KN after hyperparameter optimisation (input: all 7 conventional roughness parameters, skewness and developed interfacial ratio, only skewness, only arithmetic mean height). Mean values as red circles. Note the different y-limits for the accuracy values for the arithmetic mean height plot.

**Figure 6:**
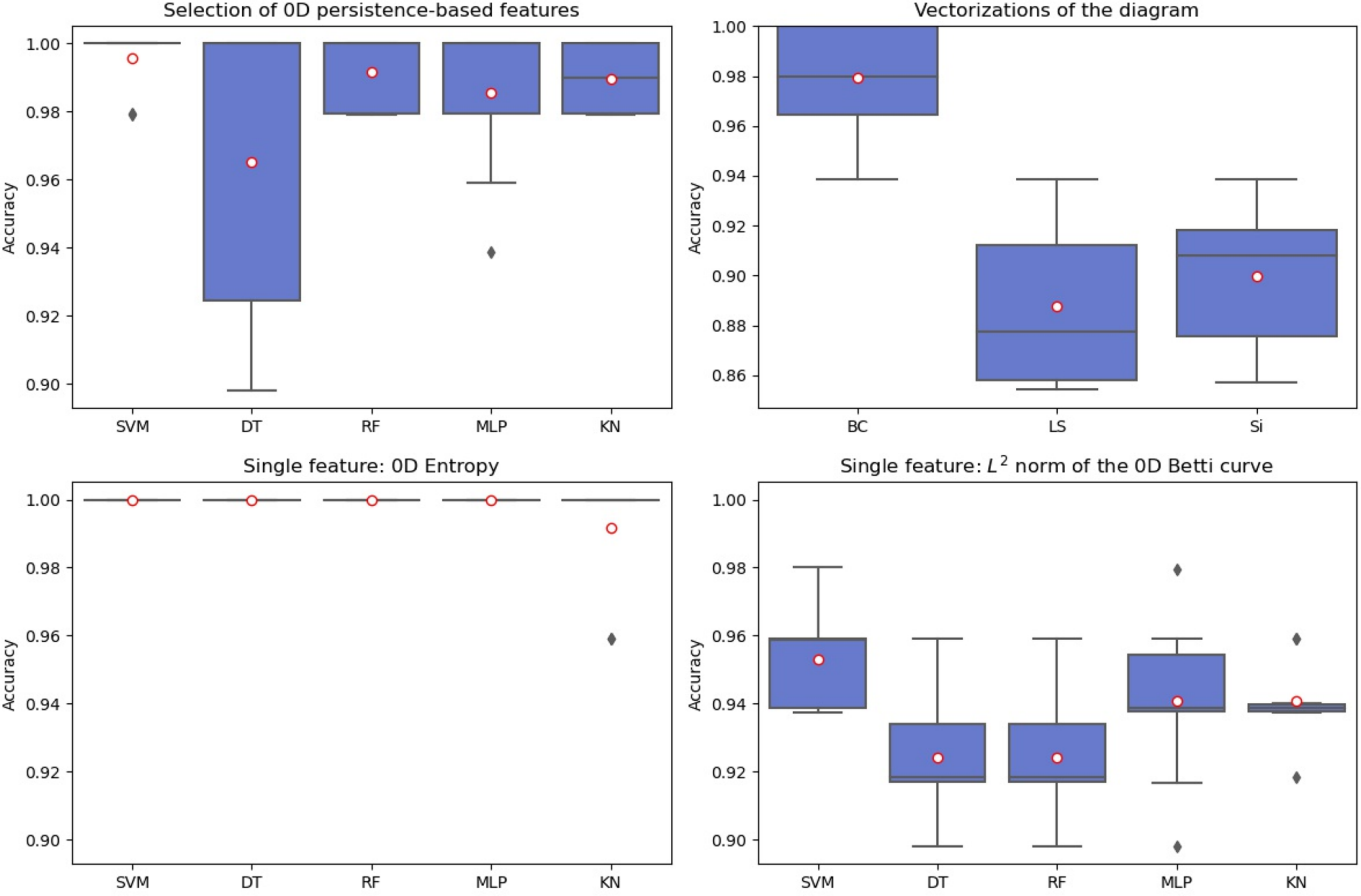
Persistence-based parameters and classification accuracy obtained for classifiers SVM, DT, RF, MLP, and KN after hyperparameter optimisation (input: all 0D persistence-based parameters, 0D entropy, ${L}^{2}$-norm of Betti curve). In addition, performance of 3 vectorizations of persistence diagrams in the upper right subplot. Mean values as red circles. Note the different y-limits for the accuracy values for the higher order vectorizations of the persistence diagrams.

Figure 5 shows the results for the classification of the test sets of the conventional parameters; Fig. 6 for the persistence-based parameters. For a direct comparison of the scalar-based conventional and persistence-based parameters, we use each feature individually for classification. In addition, the results for the training data and for the test data are shown in Table 5. It only lists the result of the best performing classifier for each parameter according to the accuracy on the test set. These classifiers are listed in the last column. Skewness shows the greatest accuracy (0.93) and F1 score (0.93) of all conventional parameters. The persistence-based parameters on the other hand show even greater accuracy (up to 1.00) in classification as well as greater F1 scores (up to 1.00). Overall, accuracy and F1 score exhibit similar rankings when considering only the scores for the test set. An indication of overfitting exists only for the root mean square gradient (Sdq) and the decision tree classifier which exhibits exceptional performance for the train set but substantially worse on the test set.

**Table 5:** Mean accuracy and mean F1 for the best performing classifier on either conventional parameters or scalar persistent-based parameters in a single feature classification.

Feature	Accuracy		F1			Obtained under classifier
	Test	Train		Test	Train	
${S}_{a}$	0.62	0.67		0.60	0.66	MLP
${S}_{dq}$	0.63	1.00		0.62	1.00	DT
${S}_{dr}$	0.64	0.71		0.63	0.69	SVM
${S}_{ku}$	0.57	0.61		0.55	0.57	SVM
${S}_{q}$	0.59	0.65		0.55	0.62	MLP
${S}_{sk}$	0.93	0.89		0.93	0.89	SVM
${S}_{z}$	0.49	0.57		0.47	0.56	SVM
${\mathrm{L}}^{2}$-Norm 0D BC	0.95	0.95		0.95	0.95	SVM
0D entropy	1.00	1.00		1.00	1.00	DT
${\mathrm{L}}^{2}$-Norm 0D LS	0.49	0.58		0.46	0.56	SVM
${\mathrm{L}}^{2}$-Norm 0D Si	0.90	0.91		0.90	0.91	SVM

To investigate possible improvements provided by a multiple feature setting, we form different input sets as specified in Table 4. Accuracy of the single feature classification in Fig. 5 decreases slightly for the collection of all conventional parameters and increases slightly for Ssk and Sdr compared to when only Ssk is applied. Persistence-based parameters shown in Fig. 6 outperform the conventional ones. Interestingly, the 0D persistence entropy shows the best accuracy when used as the sole feature during classification. This holds true for all classifiers.

The upper right plot in Fig. 6 shows the performance of persistence diagrams and their higher-dimensional vectorizations as inputs for the classification task. BC have higher accuracy (mean 0.98) compared to LS and Si as well as its ${L}^{2}$-norm. Hence, the higher dimensional TDA-based descriptors perform only slightly worse than 0D persistence entropy while containing even more relevant information about the considered surface. In the case of landscapes and silhouettes, the accuracy is worse than obtained for BC but comparable to the accuracy of all classifiers except SVM for skewness as a single feature. 0D entropy shows the overall best performance.

**Figure 7:**
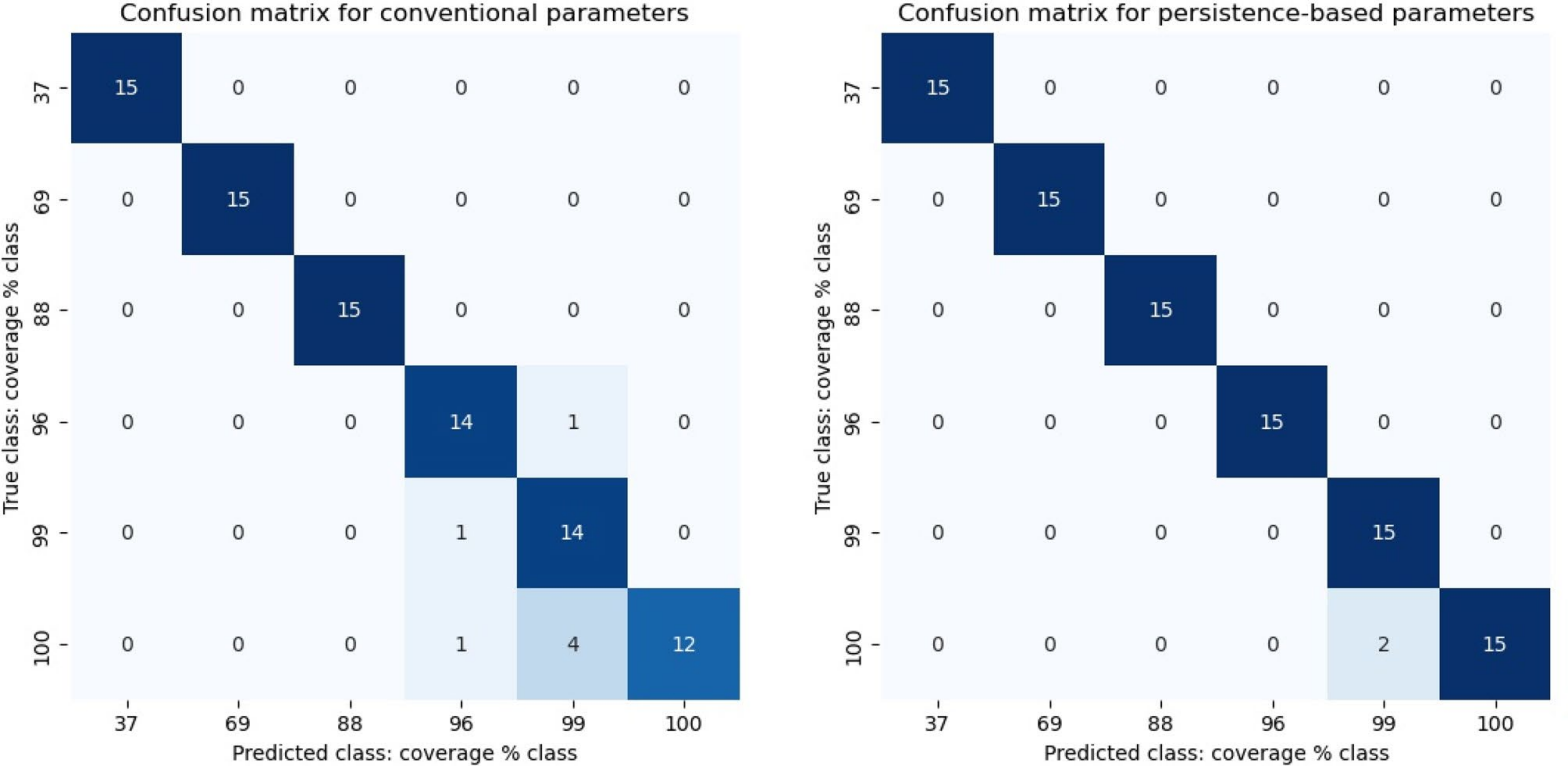
Confusion matrices for conventional and persistence-based parameters.

In engineering application, shot peening coverage can be unknown. It is of great practical interest to distinguish automatically surface samples of different coverages. This is achievable with clustering algorithms. The ToMATo clustering described in the Methods is applied to the two best performing parameters for the single feature classification; for conventional parameters those are Ssk and Sdr; for persistence-based parameters it is 0D entropy and the ${L}^{2}$-norm of the BC. In both cases, ToMATo can recover six different clusters. For the conventional parameters, we need to specify the number of clusters. For persistence-based parameters the six clusters are detected automatically. The confusion matrices for the detected labels opposed to the true labels are shown in Fig. 7. The applied clustering algorithm returns the six clusters of Table 2. In the case of the conventional parameters, seven samples are mislabelled; with incorrect labels assigned to the true 96, 99, or 100% coverage class. In the case of the persistence-based parameters, two samples from the true 100% class are mislabelled to the 99% class. The clustering results exhibit a similar pattern as before, the persistence-based parameters outperform the conventional parameters.

## Conclusions

As shown in this exploratory work, TDA techniques are a valuable extension to the toolkit given by conventional ISO surface roughness parameters. They are applied in this study for describing the evolution of surface topography in a dataset under mechanical surface treatment, Table 1. Inputs are generated based on experimentally validated code^25,28^. In our analysis, we found that persistent methods (Table 3) are outperforming standard roughness parameters (Table 2) in the task we considered even if we limit ourselves to 0D persistent parameters like entropy and BC. These two parameters are performing best at highlighting the information content of a sample as they show differences between surfaces of different coverage percentages. The vectorizations and different representations of the persistence diagrams combined with powerful and diverse machine learning algorithms, Table 4, provide a new and effective way to describe and capture a surface’s roughness in a more exhaustive way compared to the classic roughness parameters.

Using the different parameters, we use ToMATo for an unsupervised clustering task for the different parameters. While the persistence-based parameters show a better result than the conventional parameters, it should be noted that this algorithm performs well in both cases. Most importantly, this shows how TDA techniques can be built on top of existing pipelines for surface roughness.

The novelty of the work lies in proposing techniques rooted in TDA to surface roughness and showing advantages of using persistence-based parameters alongside existing conventional surface roughness parameters. Furthermore, using the unsupervised ToMATo-clustering it is highlighted how TDA can be using in conjunction with existing techniques. It should be stressed that we consider the proposed pipeline an extension of existing techniques—now formulated in the language of algebraic topology- and not a replacement of the methods highlighted in the literature for surface topography.

## Future work

In this study, we considered only conventional surface roughness parameters for a single L-filration method, namely Gaussian filtration for a particular numerical dataset of a shot peened surface. While the parameters proposed show advantages in this setting, the next steps include work to show the interconnections of TDA and existing methods for analysing surfaces topographies and the logical extension of the latter using TDA in a unified framework—both theoretically and experimentally. In the theoretical setting this includes using segmentation techniques based on Morse-Smale complexes^44^ to capture information in the merge tree^45^and combine these algorithms with the existing surface roughness parameters to give an even more comprehensive descriptor. Experimentally, performances of a wider variety of surface roughness parameters from the standards as well higher-dimensional TDA parameters should be compared and possibilities for combinations assessed. There are multiple different techniques and invariants used in TDA which led to the question of extending these to shot peened surfaces in more diverse settings and possibly to surface roughness in the context of other surface treatments. Examples are pattern recognition for the identification of materials or surface manufacturing procedures. Another advantage of considering persistent homology will be the possibility to extend the new presented framework into a more general case.

The greater precision in describing surface topography and roughness after surface treatment paves the way for more accurate engineering solutions. It will be of great interest to see results about cytocompatibility of steel surfaces^2^ and whether there is a direct relationship between cell on-growth and persistence-based roughness description. In an ideal case, these findings will translate into a better understanding of cell growth on macro 3D scaffolds^46^.

The relationship between other hyperparameters such as shot sizes and persistence-based parameters can be understood more easily using a more detailed summary provided in the persistence diagram. Incorporating such data in a persistence diagram and the persistent homology classes during construction results in an even finer comparison and is ongoing research, see^47^. In general, getting combinations of several roughness parameters in a more unified framework including TDA techniques will help to gather more insights on different surfaces. While the present study focused on the 0D persistence diagrams, 1D persistent diagrams might gain greater importance when changing other hyper parameters to consider also greater impact overlap.

## Data Availability

The calculations were made using Python code and rely on the GUDHI software package^32^. The code establishes a pipeline for the computations of surface roughness parameters and persistence-based parameters. It implements the procedure and steps demanded by ISO 25178. The analysis done in this study and the samples produced by the numerical simulation are also part of the code. The code and additional documentation can be found in the Git-Hub repository: https://github.com/janfsenge/tda_shotpeening.git.

## Abbreviations

ISO: International Organization for Standardization
0D: Zero-dimensional, dimension zero
1D: One-dimensional; dimension one
Armco: American Rolling Mill Company
BC: Persistence Betti curve
DIN: German Institue for Standarization
DT: Decision trees
FE: Finite element method
GUDHI: Geometry understanding in higher dimensions
JIS: Japanese Standards Association
KN: K-nearest neighbours vote
LDA: Linear discriminant analysis
LS: Persistence landscape
MLP: Multi-layer perceptron
PCA: Principal component analysis
RF: Random forests
Sa: Arithmetical mean height (µm)
Sdq: Root mean square gradient
Sdr: Developed interfacial area ratio
Si: Silhouettes
Sku: Kurtosis
Sq: Root mean square height (µm)
Ssk: Skewness
SVM: Support vector machine
Sz: Maximum peak to valley height (µm)
TDA: Topological data analysis
ToMATo: Topological mode analysis tool
